# “MellitusOne.”, a new technological approach to record, access, share and improve the glycemic control

**DOI:** 10.1186/1758-5996-7-S1-A56

**Published:** 2015-11-11

**Authors:** Renan Cesar Jacomassi, Lucas Leme Galastri, Ronaldo José Pineda Wieselberg, Mark Thomaz Ugliara Barone

**Affiliations:** 1 Brazilian Young Leaders Training, Brazilian Juvenile Diabetes Association (ADJ Diabetes Brazil

## Background

The medical sector has joined forces with the technological field in order to produce devices that facilitate health monitoring and diabetes management. However, this reality is not yet available to everyone. In this context, for patients who have diabetes and for their health care team (HCT), collecting and reviewing daily data are extremely important actions for effective treatment adjustments and improvements.

## Objectives

Our goal was to develop and test an affordable technological alternative for most people with diabetes who use insulin, in order to record and share data with their HCT.

## Methodology

A Microsoft® Excel® dynamic spreadsheet, called “MellitusOne.”, was created. On its first sheet personal data are inputted and in the second are the blood glucose values, insulin administered (doses and types), food consumed and corrections. On the other two sheets automated charts and graphs are presented (daily and of the last 20 days). In the tables, blood glucoses and its percentage in each category are colored according to its value (inside, below and above the target glucose). Moreover, mean, median and standard deviation values are calculated. A friendly print layout is available to print or send the reports by e-mail to the HCT. The dynamic sheet was extensively tested by the developers and then by 8 volunteers for 7 days, who answered an evaluation. Instructions were made available on the YouTube (https://youtu.be/rtj6vJFbwQU and https://youtu.be/8E5BeZQFLtI).

## Results

Seven out of eight declared that would continue using it. The main reasons raised were: easy to see glycemic variation on the graphs, which helps to keep a better glycemic control (5 answers), useful to share with the endocrinologist (2), easy to use (2), easier to take quicker decisions using it (2). Slowness on Apple computers and preference for own homemade spreadsheet was the reason why one volunteer would not continue using it. The main suggestions were: transforming it into an application (to make it more portable), increasing the fields, graphs, and automating more functions. Therefore, seven people found that “MellitusOne.” contributed to a better glycemic control.

## Conclusion

The results above, pointing towards an easy alternative to follow the glycemic control and take actions to improve it, show us that the spreadsheet “Mellitus One.” is not only useful, but also may be beneficial to this population. At the same time, systemic improvements would enhance its usability.

**Figure 1:**
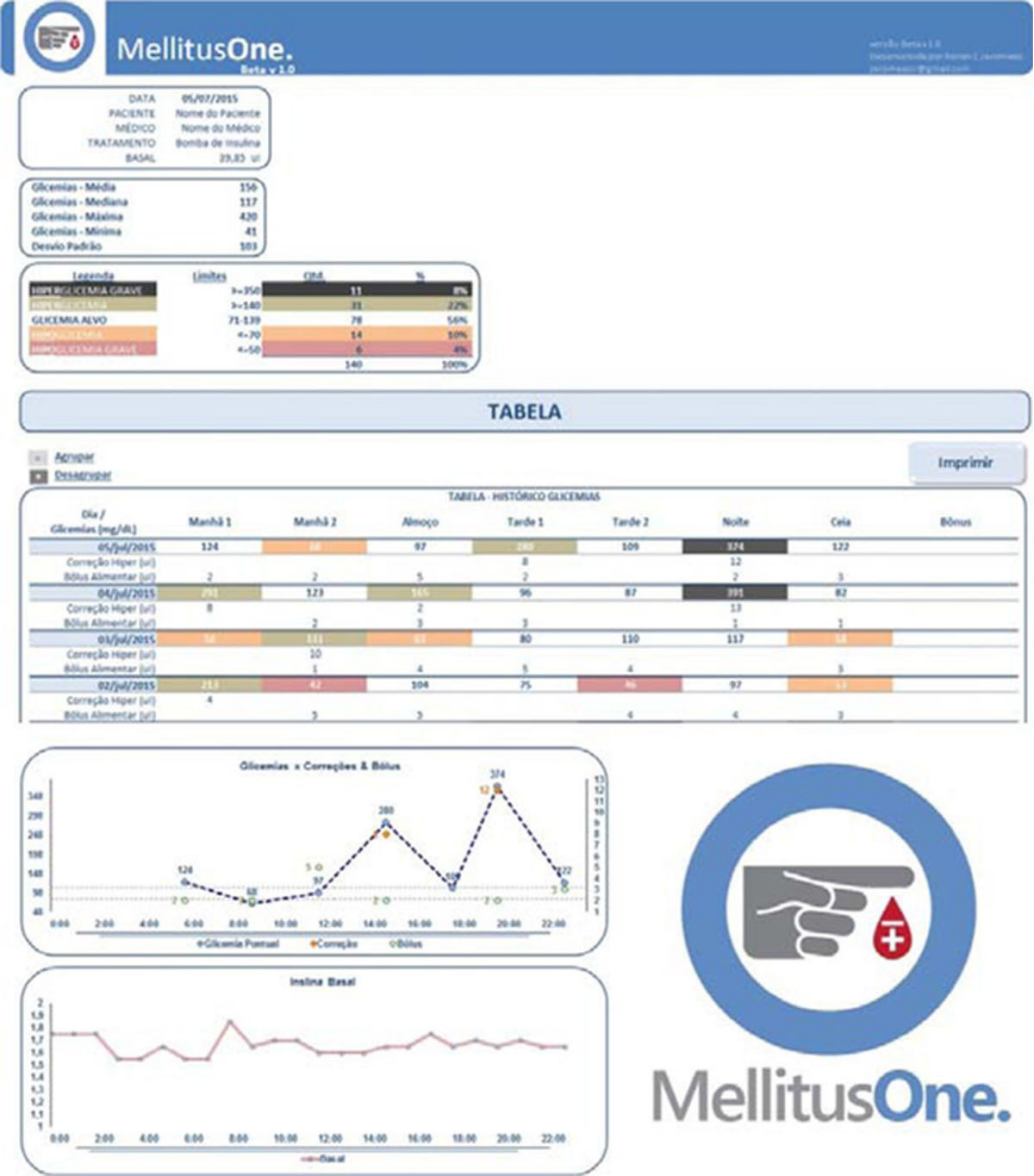
MellitusOne.

